# Evaluation of Heavy Metal Removal of Nanoparticles Based Adsorbent Using *Danio rerio* as Model

**DOI:** 10.3390/toxics10120742

**Published:** 2022-11-30

**Authors:** Antony V. Samrot, Muthiah Bavanilatha, Sivasuriyan Krithika Shree, Mahendran Sathiyasree, Jayaram Vanjinathan, Nagarajan Shobana, Rajendran Thirugnanasambandam, Chandrasekaran Kumar, Samraj Wilson, Deenadhayalan Rajalakshmi, Lawrence Xavier Noel Richard Prakash, Ram Singh Sanjay Preeth

**Affiliations:** 1 School of Bioscience, Faculty of Medicine, Bioscience and Nursing, MAHSA University, Jalan SP2, Bandar Saujana Putra, Jenjarom 42610, Malaysia; 2 Department of Biotechnology, School of Bio and Chemical Engineering Sathyabama Institute of Science and Technology, Chennai 600119, Tamil Nadu, India; 3 Department of Civil Engineering, Sathyabama Institute of Science and Technology, School of Building and Environment, Chennai 600119, Tamil Nadu, India; 4 Centre for Ocean Research (DST—FIST Sponsored Centre), MoES—Earth Science & Technology Cell, Sathyabama Institute of Science and Technology, Chennai 600119, Tamil Nadu, India; 5 Department of Botany, St. John’s College, Tirunelveli 627002, Tamil Nadu, India

**Keywords:** nanosorbent, alginate beads, SPIONs, nanotoxicity

## Abstract

Nanoparticles are potential candidates for wastewater treatment especially for the removal of heavy metals due to their strong affinity. Many biopolymers are used as adsorbents and encapsulation of nanoparticle onto them can increase their efficiency. In this study, SPIONs, alginate, and SPIONs incorporated on alginate beads have been synthesized and characterized both microscopically and spectroscopically. These were then used for the removal of chromium metal and the percentage of removal was evaluated using a batch adsorption study. The percent removal of chromium using SPIONs, alginate and alginate–SPIONs beads were recorded to be 93%, 91% and 94%, respectively. The adsorption of chromium using SPIONs and alginate–SPIONs beads followed the Tempkin isotherm, whereas adsorption of chromium metal by alginate beads was found to be homogeneous in nature and followed the Langmuir isotherm with an R^2^ value of 0.9784. An *in-vivo* study using *Danio rerio* as a model organism was done to examine the toxicity and the removal efficiency of the samples. It was observed that chromium water treated with alginate–SPIONs beads, which were removed after water treatment showed less damage to the fishes when compared to SPIONs and alginate beads treated with chromium water where the SPIONs and alginate beads were not removed after the treatment period.

## 1. Introduction

Water is one of the world’s natural resources, and its availability in its purest form is critical for all living things [1]. Effluent discharge from various industries pollutes water bodies, resulting in water pollution which could be easily monitored continuously using simple—to—use analytical technology on—site using composite sensors [2,3,4]. Effective wastewater treatment is a key requirement for a rising global economy [5]. Adsorption materials such as membranes, nanocatalysts, nanofilm—functionalized surfaces and coatings are examples of novel advanced materials that can be developed using nanotechnology for efficient wastewater cleanup procedures. Nanosorbents are one of the most ideal candidates for wastewater treatment due to the ease in utilization, high sorption capacity, etc. [6]. The use of nanoparticles in water treatment applications is restricted due to environmental contamination, potential toxicity, and safety risks. Hence, these nanoparticles can be encapsulated within a matrix material, which contributes to the treatment of water without any addition of impurities. The most commonly used biopolymers for encapsulation include polysaccharide—like alginate, cellulose, carrageenan, chitosan, starch, etc. [7,8,9,10]. Alginate beads are commonly used as a support material in numerous technologies, and they have lately been used in the removal of pollutants due to their high affinity towards heavy metal ions [11,12]. The encapsulation of alginate beads with magnetic nanoparticles, such as magnetic nanosorbents or magsorbents, helps in increasing their biosorption ability [12,13,14,15]. The beads play an important function in water treatment and are a one—of—a—kind technology for removing various organic contaminants from water [16].

These nano—adsorbent beads are good nanocomposite materials for removing heavy metals from wastewater in a continuous treatment process and are proposed to be nontoxic in nature, less expensive, to have high hydrophilicity and high efficiency [12,17]. In contrast to conventional adsorbents, nanoadsorbents are superior in ranking, status, and quality and are useful in a variety of fields, which enhances their applicability [6]. Magnetic—based adsorbents offer the key advantages of simple separation from treated water using an external magnetic field and of being reusable. For example, in the removal of phenol and p—cresol present in wastewater from the pulp and paper industry, a novel iron oxide—hydrotalcite modified with dodecylsulfate and cyclodextrin magnetic adsorbent demonstrated maximum adsorption and removed about 216.08 mg/g and 272.48 mg/g, respectively, which is greater than was reported for certain activated carbon—type and activated char adsorbents [18]. Among magnetic nanoparticles in particular, superparamagnetic Iron Oxide Nanoparticles (SPIONs) have been proposed to be an effective candidate in removal of heavy metals and also in drug delivery and imaging [19,20,21,22]. In this study, SPIONs, alginate beads, SPIONs—loaded alginate beads were prepared. These were subjected to characterization by, for example, UV—Visible Spectroscopy, Fourier Transform Infrared Spectroscopy, X-ray Crystallography, Scanning Electron Microscopy and Energy Dispersive X-ray Analysis, Vibrating Sample Magnetometer, and Zeta Potential analysis. They were then tested for removal of chromium, and the percentage of removal was examined using batch adsorption studies. Further adsorption patterns of adsorbate upon adsorbent were studied using different isotherm models. These adsorbents were used for treating water with chromium and then tested for toxicity against zebrafishes. To understand the toxicity of nanosorbent/sorbent itself, we allowed the adsorbents to be present in the water even after the treatment period in some groups.

## 2. Materials and Methods

### 2.1. Synthesis of SPIONs

SPIONs were synthesized using a chemical co—precipitation method prepared using ferric chloride (FeCl_3_) and ferrous chloride (FeCl_2_) as precursor agents: 5.4 g of ferric chloride (FeCl_3_) and 1.9 g of ferrous chloride (FeCl_2_) were mixed in 1 L of nitrogen—purged water. Then the solution was reduced by adding 40 mL of 2 M sodium hydroxide (NaOH) to it. The mixture was stirred vigorously until a black precipitate appeared. It was then centrifuged at 5000 rpm, and the pellets were collected to obtain SPIONs, which were later lyophilized [23].

### 2.2. Synthesis of Alginate Beads

25 mL of 1.2% sodium alginate (NaC_6_H_7_O_6_) solution was prepared and added dropwise to a mixture containing 10% of calcium chloride (CaCl_2_) and 1 mL of Tween 20 to form beads. Sodium alginate was transformed into beads after interacting with calcium ions. These beads were then filtered from the solution using muslin cloth [24].

### 2.3. Synthesis of Alginate–SPIONs Beads

10 mL of 1.2% sodium alginate solution was prepared, and 0.03 g of hydroxypropyl methylcellulose (HPMC) was added to it as a stabilizing agent. 125 mg of SPIONs was then added to the mixture and stirred for 40 min until the SPIONs were completely dispersed in the solution. Then the solution was added dropwise to the solution containing 10% of calcium chloride and 1 mL of Tween 20 to form beads. The beads were then filtered from the solution using muslin cloth [25].

### 2.4. Characterization of SPIONs

The UV–Visible absorption spectra of SPIONs were taken at room temperature using UV 3600 PLUS (Shimadzu, Kyoto, Japan) with a variable wavelength between 200 and 800 nm. Fourier Transform Infrared Spectroscopic experiments were carried out to check the functional groups using a IRTRACER 100 (Shimadzu, Kyoto, Japan). Scanning Electron Microscopic micrographs and elemental composition were recorded using a JSM—IT 200 (JEOL, Tokyo, Japan). The crystalline structure of the sample was determined by X-ray crystallography (PANalytical Corporation, X’Pert, Almelo, The Netherlands). The determination of the hysteresis loop was performed by Vibrating Sample Magnetometer (LakeShore Co. Ltd., Lake Shore 7400, Westerville, OH, USA) and its stability was checked using Zeta Potential analysis (ZETASIZER Nano Series ZSP, Malvern Instruments, Worcestershire, UK).

### 2.5. Characterization of Alginate Beads and Alginate–SPIONs Beads

The alginate beads and alginate–SPIONs beads were characterized using UV–Visible spectroscopy (Shimadzu, UV 3600 PLUS, Kyoto, Japan), Fourier Transform Infrared Spectroscopy (Shimadzu, IRTRACER 100, Kyoto, Japan), and crystallinity was observed using X-ray crystallography (PANalytical Corporation, X’Pert, Almelo, Netherlands).

### 2.6. Batch Adsorption Studies

The effective removal of chromium metal using SPIONs, alginate beads, alginate–SPIONs beads was done using batch adsorption studies by calculating triplicate OD values. The DPC method (1, 5—Diphenylcarbazide) was used to investigate the chromium ion adsorption [25,26]. The optimization of adsorbate concentration, adsorbent concentration, pH, and contact time was done. The adsorbate concentration (chromium metal) was initially adjusted between 1 ppm and 10 ppm, then incubated for 1 h at neutral pH. One mL of the supernatant was collected after the interaction between the samples and chromium and centrifuged for 15 min at 5000 rpm. The optimal adsorbate concentration fixed at the ppm which showed the maximum removal was observed. Then the adsorbent concentration (SPIONs, alginate beads, alginate—SPIONs beads) was altered from 0.01 g to 0.1 g/10 mL and the efficient removal was investigated under optimal adsorbate concentration. Under optimal adsorbate and adsorbent concentrations, pH was varied between 5 and 9 and checked for the removal efficiency. Similarly, effective removal of adsorbate by adsorbent was examined by altering the contact time between 30 min and 2 h 30 min under the optimized adsorbate, adsorbent and pH conditions. After optimization, 1 mL of supernatant was treated with 30 mL of 2N H_2_SO_4_, which produces Cr^6+^ dissociation, and further treated with 0.5% of diphenyl carbazide, which gives a reddish—violet color by binding to Cr^6+^. It was then spectroscopically measured at 540 nm [26]. The formula used to calculate the adsorption removal is given below:

% Removal = [(Initial concentration–Final concentration)/(Initial concentration)] × 100

### 2.7. Isotherm Studies

The adsorption pattern and the mechanism of adsorbate removal by adsorbent was examined using four types of isotherm models—Langmuir, Freundlich, Tempkin and Dubinin—Radushkevich (D—R) isotherm. A graph between 1/qe and 1/Ce was plotted for the Langmuir isotherm, ln qe vs. ln Ce for the Freundlich, the Tempkin isotherm was investigated by plotting qe vs. ln Ce, and D—R was determined by plotting between ε^2^ and ln qe in order to determine the adsorption pattern, where Ce is the equilibrium concentration of adsorbate in mg/L, qe is the amount of adsorbate adsorbed upon the adsorbent, and ε^2^ is the adsorption potential based on the Polanyi potential.

### 2.8. In-Vivo Study to Determine Removal of Chromium

#### 2.8.1. Collection and Maintenance of Zebrafish

Male *Danio rerio* were purchased from Tarun fish farm, Chennai and kept in a glass aquarium filled with water with sufficient and continuous aeration. The fishes were introduced into the test environment after three to four days of acclimating to the new circumstances. The water was changed once a week and dry food fed twice a day. The initial pH of the water was at 7.2 ± 0.3 [27].

#### 2.8.2. *In-Vivo* Study Using Zebrafish as Model

An *in-vivo* study for the effective removal of chromium using SPIONs, alginate beads, and alginate–SPIONs beads was done against *Danio rerio*. Test 1 with 1 ppm of chromium water was used to test the survivability of zebrafishes. In Test 2, 7 g of synthesized SPIONs were added to 5 L of water. In Test 3, 7 g of SPIONs were mixed with 1 ppm chromium water and was not removed. In Test 4, SPIONs—treated water was used, whereby 7 g of SPIONs were added to 1 ppm of chromium water and the SPIONs were withdrawn after 1 h of interaction before introducing the fishes. In Test 5, 7 g of synthesized SPIONs were put into the water, and after 1 h of interaction they were removed. In Test 6, SPIONs—encapsulated alginate beads were added to 1 ppm of chromium water and not removed. In Test 7, SPIONs—encapsulated alginate beads were put into chromium water and removed after 1 h of interaction. In Test 8, 7 g of alginate beads were introduced to 1 ppm of chromium water and were not removed. Ten fishes were allowed inside the aquarium which contained the aforementioned conditions. All the fishes were fed regularly, as mentioned.

#### 2.8.3. Histology Studies

The damage that occurred in the fishes on exposure to the chromium water and SPIONs was analyzed using histology studies. The fishes were taken from each test on the 5th and 10th days and were put into 10% of formaldehyde solution for analysis. The organs of the fish—eyes and gills—were then subjected to Hematoxylin and Eosin (H&E) staining and examined using a fully inverted microscope (Leica DMI6000 B, Leica Microsystems, Stockach, Germany).

#### 2.8.4. Inductively Coupled Plasma—Mass Spectrometry (ICP—MS)

The uptake of chromium and iron metals by the fish was examined on the 5th and 10th days using ICP—MS (Inductively Coupled Plasma Mass Spectrometry) through the microwave digestion method. The whole test fish was taken from the aquarium and immersed in 5 mL of concentrated nitric acid for 15 min. A few drops of concentrated hydrofluoric acid were then added and microwaved until fully digested. A clear solution was obtained by centrifugation which was then taken and kept at 4 °C and then analyzed using Agilent 7700x—ICP—MS (Agilent Technologies, Mulgrave, VIC, Australia).

## 3. Results and Discussion

### 3.1. Characterization of SPIONs, Alginate Beads and SPIONs—Coated Alginate Beads

#### 3.1.1. UV—Visible Spectroscopy

**Figure 1:**
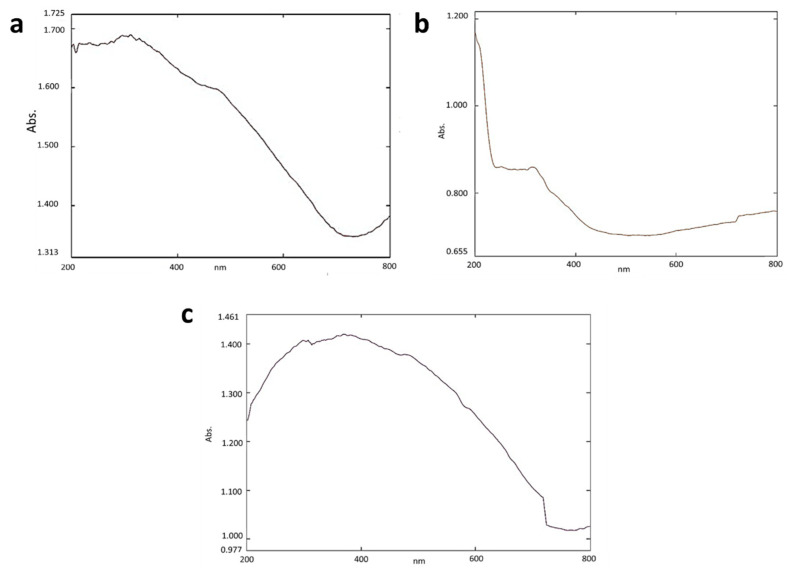
UV—Vis analysis of (**a**) SPIONs (**b**) Alginate beads (**c**) Alginate–SPIONs microspheres.

The peak at the range between 250 and 300 nm shows the presence of iron oxide nanoparticles, and the peak between 200 to 400 nm shows the light absorption of the alginate beads (Figure 1a,b). A broad peak from 250 to 400 nm shows the presence of SPIONs indicating the encapsulation of SPIONs into the alginate beads (Figure 1c). Lam et al. [28] reported that absorption of light at 200 to 400 nm shows the presence of alginate beads. The maximum absorption at 250 to 300 nm indicates the characteristic peak for the SPIONs [29].

#### 3.1.2. Fourier Transform Infrared Spectroscopy (FTIR)

**Figure 2:**
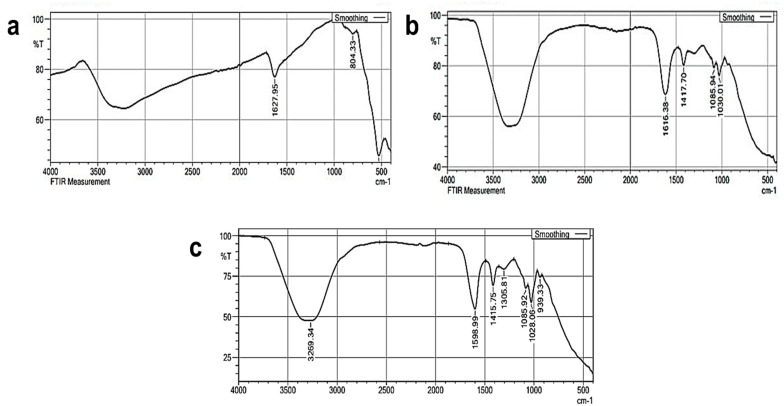
FTIR analysis of (**a**) SPIONs (**b**) Alginate beads (**c**) Alginate–SPIONs microspheres.

The functional groups present in the SPIONs, alginate beads and SPIONs—coated alginate beads were analyzed using Fourier Transform Infrared Spectroscopy (FTIR) (Figure 2). The shift of the C=O bond due to the formation of an ionic bond between calcium chloride and sodium alginate was found at the peak at 1616 cm^−1^. A peak around 3400 to 3300 cm^−1^ was due to hydroxyl groups and is seen in all the samples. A large peak seen in Figure 2b,c at 630–550 cm^−1^ depicts the presence of Fe–O vibrations of SPIONs [30]. Matos et al. [31] reported that peaks at 3500 cm^−1^, 453 cm^−1^, and 586 cm^−1^ showed the presence of an OH group on the surface of SPIONs and the presence of the Fe–O bond of SPIONs, respectively. In another study, the presence of Fe_3_O_4_ particles in the Alg—Ce magnetic bead was depicted by the presence of a novel absorption band at 561 cm^−1^ [32].

#### 3.1.3. X-ray Diffraction (XRD)

**Figure 3:**
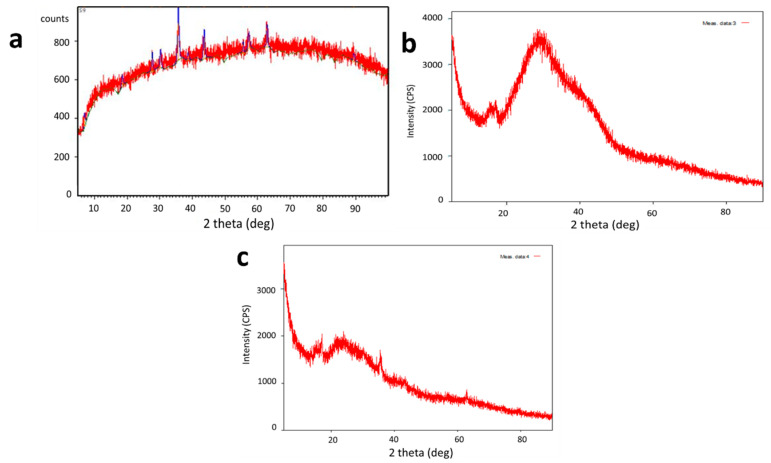
XRD analysis of (**a**) SPIONs (**b**) Alginate beads (**c**) Alginate–SPIONs microspheres.

The crystalline structure of the samples was examined through X-ray diffraction (XRD) (Figure 3). The crystal peaks of 2θ at 28.8°, 34.2°, 38.6°, 44.5°, 61.7° and 67.4° depict the crystallinity of synthesized SPIONs. The irregular peaks of alginate beads revealed that they were amorphous in nature. The encapsulation of SPIONs was revealed by its crystallite structure on alginate beads through the regular peaks at 9.04°, 13.78°, 38.93°, and 62.21°. The spinel structure of the SPIONs was depicted by the multiple peaks at 2θ of 18.25°, 30.06°, 35.63°, 43.48°, 53.78°, 57.33°, and 63.11° [33]. Lilhare et al. [34] reported that sharp peaks of calcium alginate entrapped on methionine—functionalized magnetic nanoparticles indicate that the particles are crystalline and tiny and correspond to the planes of a Fe_3_O_4_ face—centered cubic structure.

#### 3.1.4. Scanning Electron Microscopy and Energy Dispersive X-ray Analysis

**Figure 4:**
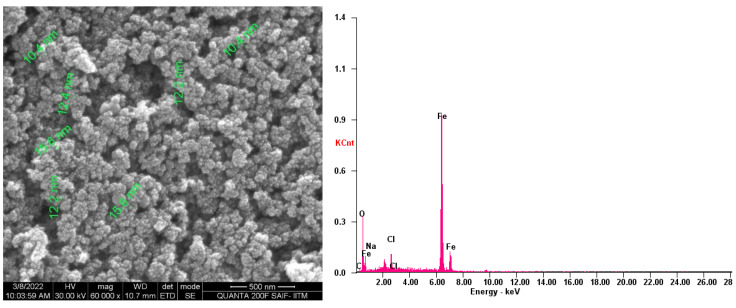
SEM and EDX analysis of SPIONs.

The surface morphology and the elementary composition of the SPIONs were examined using Scanning Electron Microscopy (SEM) and Energy Dispersive X-ray Analysis (EDX) (Figure 4). The size of the synthesized SPIONs were 10–15 nm in size, and the major compounds like Fe, O and minor compounds such as Na and Cl were observed through EDX. Baykal et al. [35] reported sizes of SPIONs of around 12 nm. There are reports on SPIONs of size between 14.53 nm and 20.54 nm with the elemental composition of Fe and O [36].

#### 3.1.5. Zeta Potential Analysis

**Figure 5:**
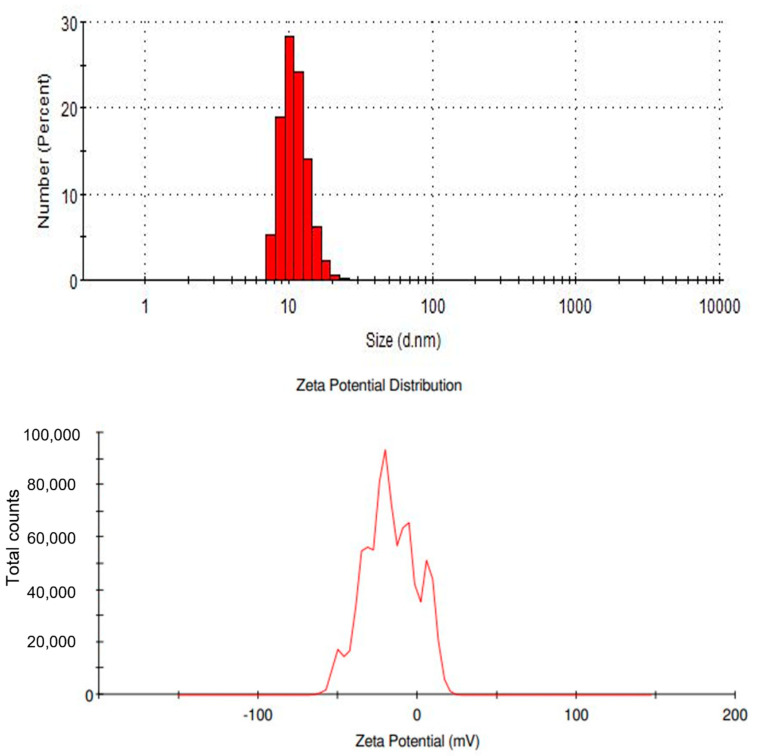
Zeta potential analysis of SPIONs.

The charges of the SPIONs were observed using Zeta Potential analysis (Figure 5). The peaks of the analysis showed that the synthesized SPIONs carried negative charges. Unlike iron nanoparticles that exhibited a low surface charge, the synthesized SPIONs used in this study had a much higher Zeta Potential; Khatami et al. [37] also produced SPIONs using Stevia plant with a magnitude of —41.1 mV [37]. In contrast, Reczyńska et al. [38] also reported the charge of SPIONs to be 19.6 ± 0.8 mV.

#### 3.1.6. Vibrating Sample Magnetometer

**Figure 6:**
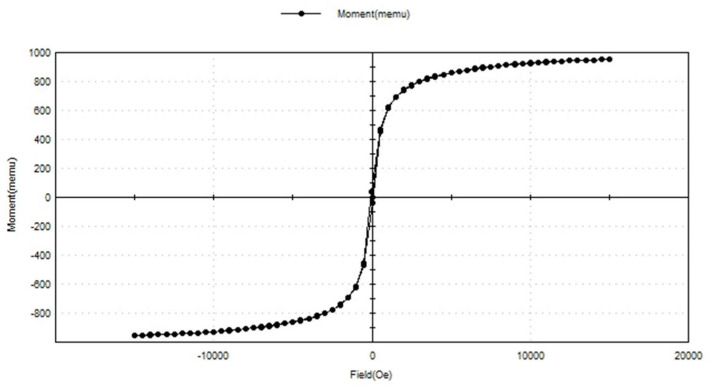
VSM analysis of SPIONs.

The magnetism of the SPIONs was examined using a vibrating sample magnetometer (VSM) (Figure 6). The hysteresis loop of the synthesized SPIONs showed the superparamagnetic nature of the SPIONs. Raees et al. [39] reported that saturation magnetization of SPIONs was found to be 71.82 emu/g, indicating a very high saturation magnetization and superparamagnetic behavior. The SPIONs produced in this study also showed a high saturation magnetization.

### 3.2. Batch Adsorption Studies

Batch adsorption experiments on the removal of chromium utilizing SPIONs, alginate beads, and alginate–SPIONs beads were studied using the DPC method. The conditions such as adsorbate concentration, adsorbent concentration, pH, and contact time were all optimized.

#### 3.2.1. Removal of Chromium Using SPIONs

**Figure 7:**
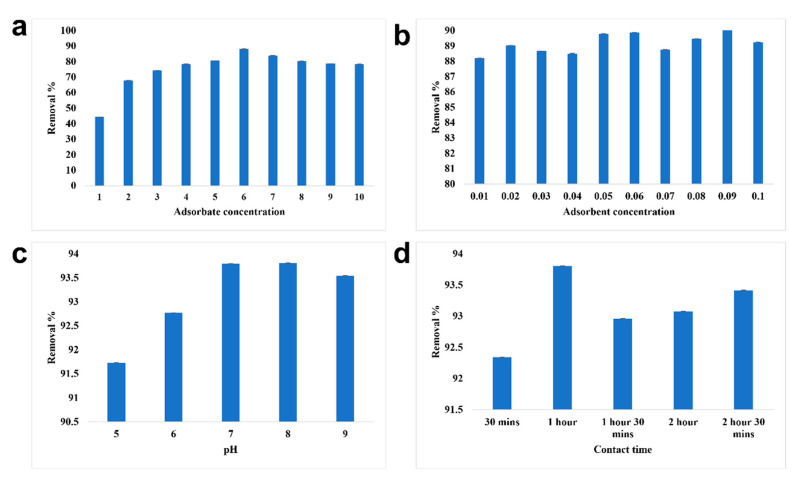
Batch adsorption study of SPIONs. (**a**) Optimization of adsorbate concentration, (**b**) Optimization of adsorbent concentration, (**c**) Optimization of pH, (**d**) Optimization of contact time.

The optimized condition for the effective removal of chromium from water was studied (Figure 7). The maximum removal of chromium was observed at 6 ppm at a removal percentage of 88.19%. With the adsorbate concentration, the adsorbent concentration was optimized; 0.09 g/10 mL of SPIONs was observed to remove about 90.88%. At pH of 8, 6 ppm of chromium was removed efficiently, at 0.09 g/10 mL, with the increased removal of 1 h. The interaction time between the adsorbate and adsorbent plays a major role in the removal. The optimized contact time was examined to be 1 h with a removal efficiency of 93.8%. Farhana et al. [19] reported that SPIONs with impregnated activated carbon showed a removal percentage of nearly 99.7% with the optimized adsorbate concentration of 1 ppm and adsorbent concentration of 10 g/L. It was revealed that as the concentration of chromium increases, iron leaching was high, as there is no active site readily available for the adsorption reaction to take place. The electrostatic interaction between the adsorbent surface (SPIONs) and anion (chromates) is the driving force for the adsorption process [9,19].

#### 3.2.2. Removal of Chromium Using Alginate Beads

**Figure 8:**
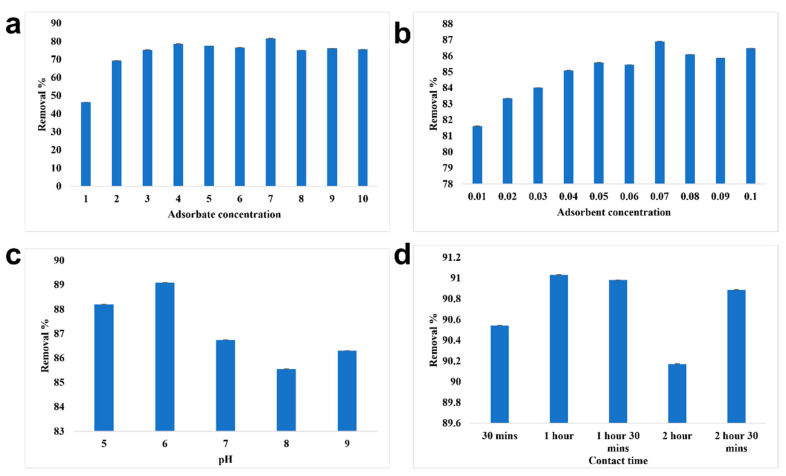
Batch adsorption study of alginate beads. (**a**) Optimization of adsorbate concentration, (**b**) Optimization of adsorbent concentration, (**c**) Optimization of pH, (**d**) Optimization of contact time.

The adsorbate concentration of 7 ppm was removed efficiently by alginate beads with a removal percentage of 81.61% (Figure 8). Under the optimized adsorbate concentration, the maximum removal of chromium was recorded at 0.07 g/10 mL of adsorbent concentration. Using 0.07 g/10 mL of alginate beads, 7 ppm of chromium was efficiently removed at pH 6 and the contact time was found to be 1 h. The overall removal percentage of chromium using alginate beads was recorded to be 90.98%. Babu et al. [40] reported that red mud doped in calcium alginate beads with hydrazine sulphate activation had the ability to remove 25 mg/L of lead with 100% efficiency and that the removal efficiency decreased with the increase in concentration. The adsorbent concentration of 2.0 g/100 mL removed nearly 91.5%. As lead is cationic in nature, pH 6 was found to be the optimized condition for the efficient removal of lead using alginate beads. As the pH increases, the removal percentage changes due to the imposition of a negative charge on the surface of the adsorbate. The optimized contact time was recorded to be 180 min and higher rate of adsorption was observed initially due to greater vacant site availability in the adsorbent.

#### 3.2.3. Removal of Chromium Using Alginate–SPIONs Beads

**Figure 9:**
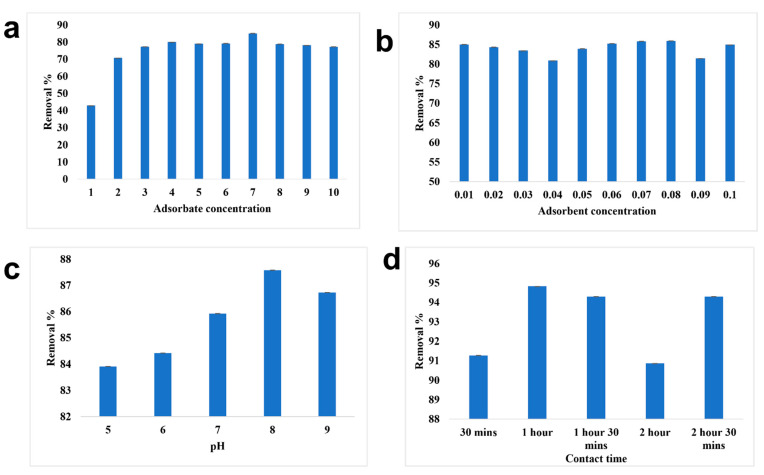
Batch adsorption study of alginate–SPION microspheres. (**a**) Optimization of adsorbate concentration, (**b**) Optimization of adsorbent concentration, (**c**) Optimization of pH, (**d**) Optimization of contact time.

The removal of chromium using alginate–SPIONs beads was found to be higher than that of SPIONs and alginate beads (Figure 9). 7 ppm of chromium were removed efficiently by the alginate–SPIONs beads with an efficiency of 85.03% due to the combined activity of alginate beads with that of SPIONs. The optimized adsorbent concentration was evaluated to be 0.08 g/10 mL with a removal percentage of 85.92%. The optimized pH and contact time under optimized adsorbate and adsorbent concentration was found to be 8 pH and 1 h, respectively. The maximum removal percentage of chromium by alginate–SPIONs beads was evaluated to be 94.84%. Idris et al. [41] revealed the magnetic alginate beds has the ability to removal heavy metals such as lead from water. The optimized adsorbent concentration was reported to be 50 mg/g with the adsorbate concentration of 400 mg/L. It was reported that the removal efficiency increased with the increase in pH until reaching neutral pH. The adsorption mechanism was observed to be of two stages: rapid adsorption on adsorbate—adsorbent contact, and lower adsorption until equilibrium was reached as the vacant spaces became fewer.

#### 3.2.4. Isotherm Studies

**Figure 10:**
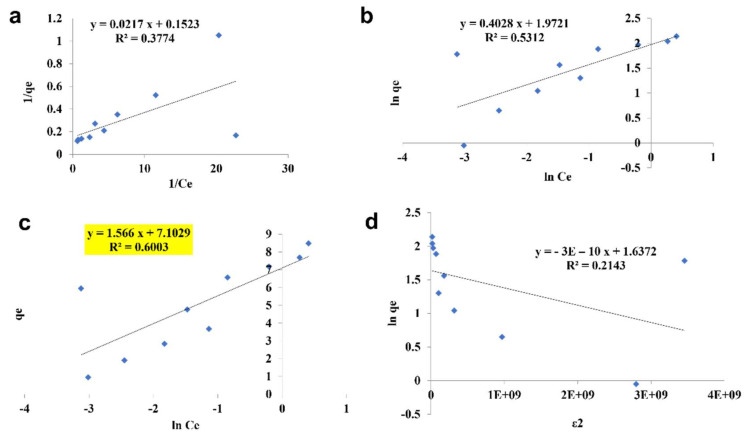
Isotherm study of SPIONs. (**a**) Langmuir Isotherm, (**b**) Freundlich Isotherm, (**c**) Tempkin Isotherm, (**d**) D—R Isotherm.

**Figure 11:**
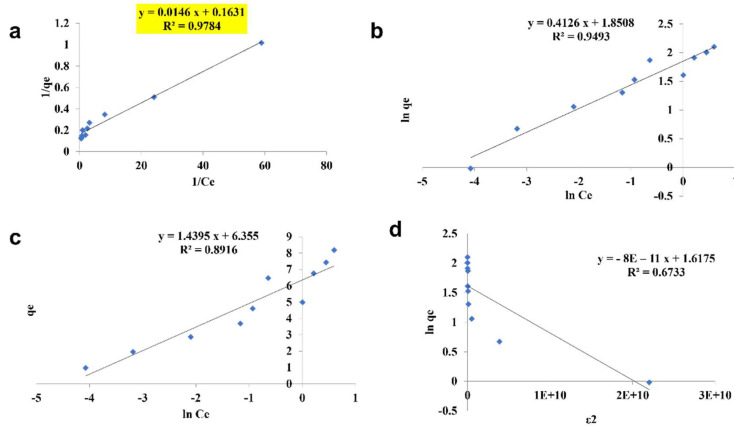
Isotherm study of alginate beads. (**a**) Langmuir Isotherm, (**b**) Freundlich Isotherm, (**c**) Tempkin Isotherm, (**d**) D—R Isotherm.

**Figure 12:**
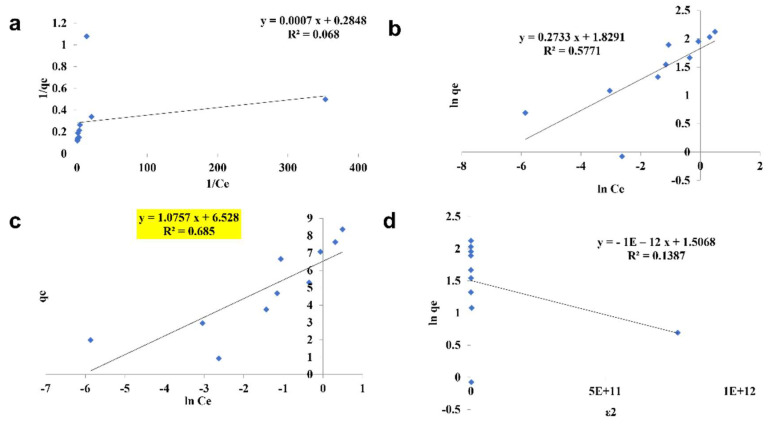
Isotherm study of alginate–SPIONs microspheres. (**a**) Langmuir Isotherm, (**b**) Freundlich Isotherm, (**c**) Tempkin Isotherm, (**d**) D—R Isotherm.

The pattern by which the adsorbent adsorbs the adsorbent is studied using four kinds of isotherm model: the Langmuir isotherm, the Freundlich isotherm, the Tempkin isotherm, and the D—R isotherm (Figure 10, Figure 11 and Figure 12). The best—fit isotherm model was identified by the comparison of correlation coefficient (R^2^) values of each isotherm (Highlighted in yellow). The adsorption of chromium using SPIONs and alginate–SPIONs beads was found to be ionic in nature, as it followed the Tempkin isotherm with an R^2^ of 0.6003 and 0.7215. Homogeneous adsorption of chromium on alginate beads was recorded, as it followed the Langmuir isotherm with the highest R^2^ value of 0.9784. The removal of chromium using SPIONs was found to follow the Freundlich isotherm, with the highest R^2^ of 0.9228 [31]. Bilici et al. [42] reported that adsorption of oil contaminant from wastewater by calcium alginate beads functionalized with sodium dodecyl sulphate (SDS) was found to follow the Langmuir isotherm with a regression coefficient 0.9999. Further, the removal of lead using magnetic nano—sorbent was found to be heterogenous in nature, following the Freundlich isotherm [43].

### 3.3. Toxicity Studies

#### 3.3.1. Histology Studies

**Figure 13:**
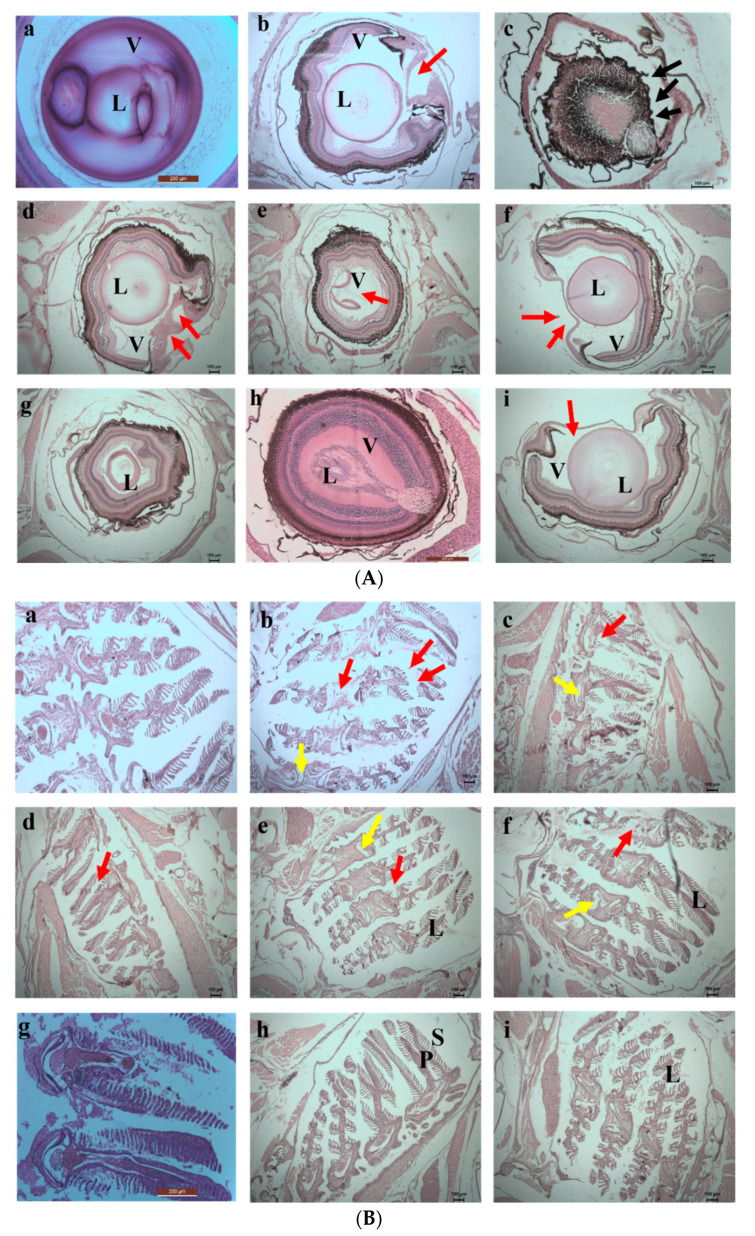
(**A**) 5th day. Histology images of eyes of zebrafish exposed to (**a**) Control (**b**) **Test 1**—Chromium metal in water (**c**) **Test 2**—SPIONs in water (**d**) **Test 3**—Chromium metal with water in SPIONs (**e**) **Test 4**—Chromium metal in water with SPIONs removed after interaction for 1 h (**f**) **Test 5**—Chromium metal in water with alginate–SPIONs microspheres (**g**) **Test 6**—Chromium metal in water with alginate–SPIONs microspheres removed after interaction for 1 h (**h**) **Test 7**—Chromium metal in water with alginate beads (**i**) **Test 8**—Chromium metal in water with alginate beads removed after interaction for 1 h. (Red arrow denotes erosion; L—lens, V—Vitreous). (**B**) 5th day. Histology images of gills of zebrafish exposed to (**a**) Control (**b**) **Test 1**—Chromium metal in water (**c**) **Test 2**—SPIONs in water (**d**) **Test 3**—Chromium metal with water in SPIONs (**e**) **Test 4**—Chromium metal in water with SPIONs removed after interaction for 1 h (**f**) **Test 5**—Chromium metal in water with alginate–SPIONs microspheres (**g**) **Test 6**—Chromium metal in water with alginate–SPIONs microspheres removed after interaction for 1 h (**h**) **Test 7**—Chromium metal in water with alginate beads (**i**) **Test 8**—Chromium metal in water with alginate beads removed after interaction for 1 h. (Red arrow denotes erosion, yellow arrow denotes the intestinal space; Black arrow denotes deposition (P—Primary Lamella, S—Secondary Lamella, L—Lamella).

The damage caused by the exposure to the chromium and the adsorbents was evaluated by histology study. Histology images taken on the 5th day are shown in Figure 13a,b. On the 5th day of exposure, the direct interaction of fishes with the chromium water caused damage to the lens of the eyes, and complete distortion occurred in the vitreous region of the eyes. In the fishes exposed to the water containing SPIONs there were also major impacts. Huge accumulations of SPIONs in the pupil, iris and the lens region of the eyes were observed. Moreover, distortion of the lamellar epithelium in the gill region and the hepatic lobules of the liver were noted. However, less damage was observed in the SPIONs—treated chromium water, and adsorbent was removed. The damage in the retinal ganglion cell layer and deformation of the gill sacs and filaments were noted in Tests 5 and 7. In comparison to these tests, less damage to the fishes was observed in the chromium water treated with alginate beads/alginate–SPIONs beads, and these adsorbents were removed after 1 h.

**Figure 14:**
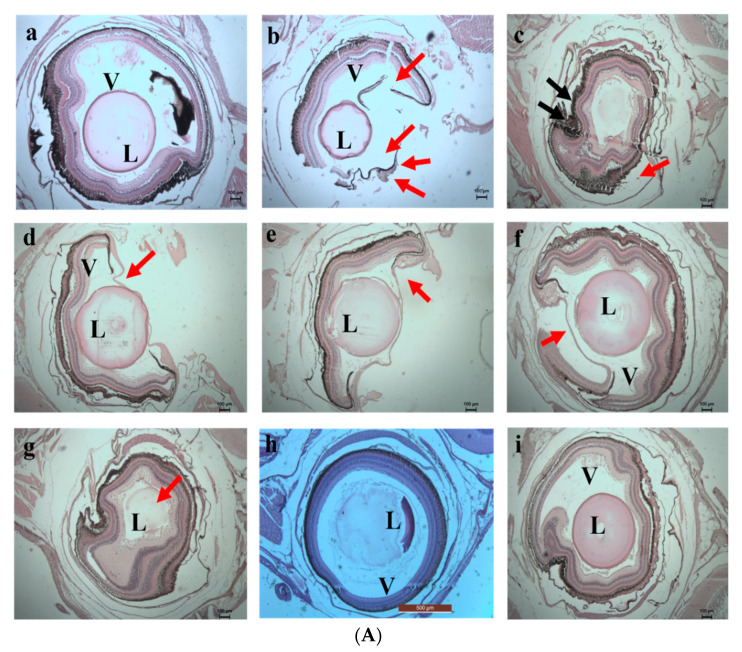

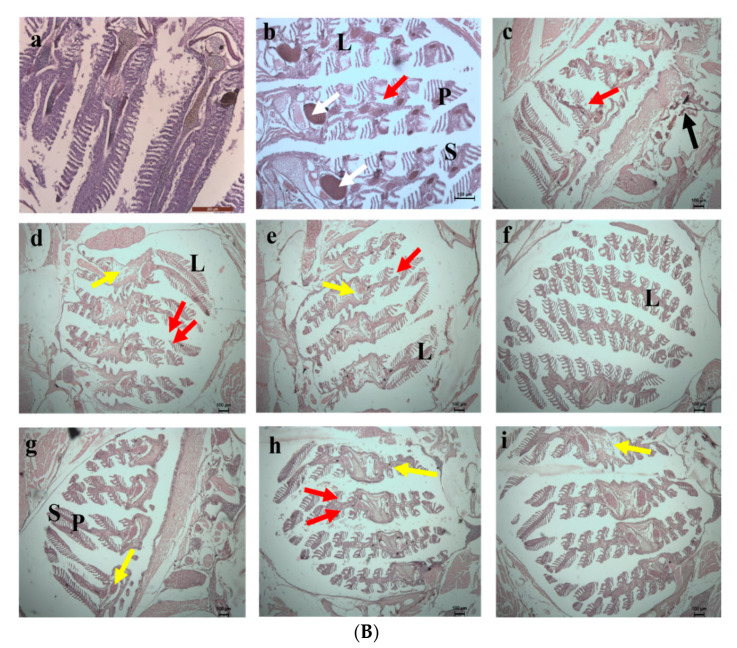
(**A**) 10th day. Histology images of eye of zebrafish exposed to (**a**) Control (**b**) **Test 1**—Chromium metal in water (**c**) **Test 2**—SPIONs in water (**d**) **Test 3**—Chromium metal with water in SPIONs (**e**) **Test 4**—Chromium metal in water with SPIONs removed after interaction for 1 h (**f**) **Test 5**—Chromium metal in water with alginate–SPIONs microspheres (**g**) **Test 6**—Chromium metal in water with alginate–SPIONs microspheres removed after interaction for 1 h (**h**) **Test 7**—Chromium metal in water with alginate beads (**i**) **Test 8**—Chromium metal in water with alginate beads removed after interaction for 1 h. (Red arrow denotes erosion; L—Lens, V—Vitreous). (**B**) 10th day. Histology images of gills of zebrafish exposed to (**a**) Control (**b**) **Test 1**—Chromium metal in water (**c**) **Test 2**—SPIONs in water (**d**) **Test 3**—Chromium metal with water in SPIONs (**e**) **Test 4**—Chromium metal in water with SPIONs removed after interaction for 1 h (**f**) **Test 5**—Chromium metal in water with alginate–SPIONs microspheres (**g**) **Test 6**—Chromium metal in water with alginate–SPIONs microspheres removed after interaction for 1 h (**h**) **Test 7**—Chromium metal in water with alginate beads (**i**) **Test 8**—Chromium metal in water with alginate beads removed after interaction for 1 h. (Red arrow denotes erosion, Yellow arrow denotes the intestinal space; Black arrow denotes deposition; White arrow denotes Lipofuscin (P—Primary Lamella, S—Secondary Lamella, L—Lamella).

On the 10th day of exposure, Test 1 showed increased damage in the eyes and gill regions (Figure 14a,b). Higher accumulation of SPIONs was observed in the lens of the eyes. The degradation of the lamella and the secondary lamella of the gills were observed in fishes exposed to chromium water treated with SPIONs (and SPIONs not removed). The fishes exposed to chromium water treated with alginate—SPIONs and then removed showed no damage in the eye lens, and lesser damage to the gill filament were recorded. Aldavood et al. [44] reported that the exposure of zebrafish embryos to heavy metals such as cadmium caused a decrease in myosin protein expression and affected the function and development of muscle. A study which investigated the removal efficiency of chromium using graphene oxide revealed damage in the skeletal muscles, misfolding of proteins and oxidative stress in zebrafishes and its embryos [45]. It was revealed that magnetite—based nanocomposites imposed a lower toxicity on zebrafish with less variation in shape, color and behavior [46,47].

#### 3.3.2. Inductively Coupled Plasma—Mass Spectrometry (ICP—MS)

**Figure 15:**
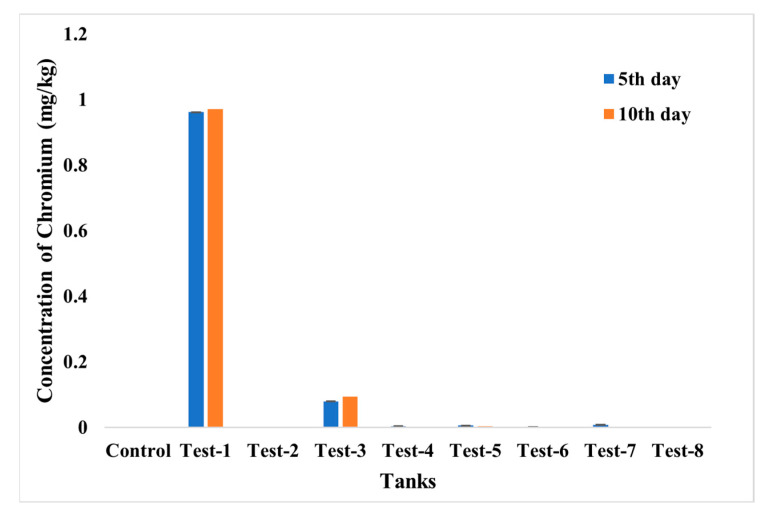
ICP—MS analysis for chromium accumulation analysis in control; **Test 1**—Chromium water; **Test 2**—SPIONs in water; **Test 3**—Chromium water with SPIONs; **Test 4**—Chromium water treated with SPIONs; **Test 5**—Chromium water with alginate–SPIONs microspheres; **Test 6**—Chromium water treated with alginate–SPIONs microspheres; **Test 7**—Chromium water with alginate beads; **Test 8**—Chromium water treated with alginate beads.

**Figure 16:**
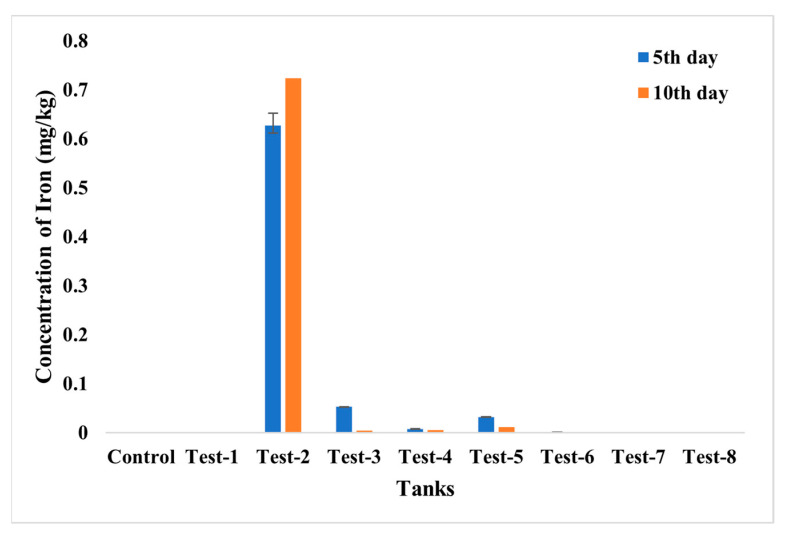
ICP—MS analysis for iron accumulation analysis in control; **Test 1**—Chromium water; **Test 2**—SPIONs in water; **Test 3**—Chromium water with SPIONs; **Test 4**—Chromium water treated with SPIONs; **Test 5**—Chromium water with alginate–SPIONs microspheres; **Test 6**—Chromium water treated with alginate–SPIONs microspheres; **Test 7**—Chromium water with alginate beads; **Test 8**—Chromium water treated with alginate beads.

The accumulation of chromium and the iron in fish was evaluated using ICP—MS analysis (Figure 15 and Figure 16). From the analysis, the highest accumulation of chromium was observed only in Test 1 at a concentration of 0.96–0.97 mg/kg, then in Test 3, whereas fishes in other tests were found to have negligible concentrations of chromium accumulation. The accumulation of iron was also estimated, and it was recorded in fishes in Tests 3 to 7. The highest concentration, of 0.71 mg/kg, was recorded in the fish grown in Test 3 and may have arisen from dispersion of SPIONs from alginate beads.

## 4. Conclusions

SPIONs, alginate beads and alginate–SPIONs beads were successfully synthesized, characterized and evaluated for the removal of chromium from contaminated water. It was concluded that the removal of chromium by alginate–SPIONs beads is higher than that of SPIONs and alginate beads with the optimized adsorbate and adsorbent concentration of 7 ppm and 0.08 g/10 mL, respectively, at pH 8 with a contact time of 1 h. The toxicity study also revealed that chromium water treated with the alginate–SPIONs beads (where the adsorbents were removed) imposed less damage on zebrafish (*Danio rerio*), and therefore it may be one of the better and lower—cost biosorbents for removing heavy metals. The presence of absorbents was found to cause lethal impacts to the animals; therefore, it is suggested not to allow the adsorbents into the treatment system following the treatment process.

## Data Availability

The data used to support the findings of this study are included in the article. Should further data or information be required, these are available from the corresponding author upon request.
